# Osmolyte effects: revisiting solubility measurements, accessible surface area categorization, and language for communicating with a broad audience

**DOI:** 10.7717/peerj.20623

**Published:** 2026-03-27

**Authors:** Jonathan G. Cannon

**Affiliations:** Department of Natural Sciences, Middle Georgia State University, Cochran, GA, United States of America

**Keywords:** Osmolytes, Transfer model, Solute partitioning, Solubility, Accessible surface area, Osmophobic effect, Hydrophobic effect, Teaching biochemistry

## Abstract

The osmophobic effect has been an accessible phrase to introduce biochemists to ideas of how osmolytes affect biochemical processes. It was built by analogy with the hydrophobic effect, central to protein folding, inspired by the initial observation that urea primarily interacts with the backbone of proteins, and less significantly with side chains. First, the author and his students revisited an experiment underpinning the original formulation of the osmophobic effect. New solubility measurements for glutamate in the presence of glycine betaine indicate that these molecules interact extremely unfavorably, in contrast to the small, favorable interaction previously reported from a solubility study, and in agreement with the interaction reported from vapor pressure osmometry measurements. This error in solubility measurements, in combination with numerous experimental findings published by other researchers, confirms a significant role for side chain-glycine betaine interactions, particularly with anionic oxygen. Second, the author compares two accessible surface area categorizations: dividing protein surface into backbone and side chains (used in the group transfer free energy model), and dividing molecules into atomic surface types (used in the solute partitioning model). Accessible surface areas of the Trp-cage peptide are used to illustrate the greater computational simplicity and generalizability of an atom based partitioning model over a chemical group based model. Third, the author offers a perspective on communicating osmolyte effects to a broader audience. Combining the widespread observation that osmolyte side-chain interactions are very significant, with the illustrated advantages of an atom-based surface area model, the author suggests reframing and reclaiming the term “osmophobic effect”. By separating the phrase “osmophobic effect” from its roots emphasizing protein backbone interactions influencing protein folding, and expanding it to include osmophobic and osmophilic interactions with proteins and other biological molecules, researchers and educators will foster easier understanding of osmolyte effects through analogy with the hydrophobic effect. Further, this analogy could be strengthened through application of increasingly accurate osmophobic/osmophilic models that rely on atomic surface types—like the hydrophobic effect relies on hydrophobic surfaces rather than specific side chains, and is valuable for understanding lipid aggregation and nucleic acid structure formation in addition to protein folding.

## Introduction

### Osmolyte effects

As we introduce new students to the function of proteins, nucleic acids, and other biological molecules, biochemistry courses emphasize the relationship between structure and function. We proceed from well-understood, well-defined molecular structures with uncomplicated reaction kinetics in order to illustrate powerful biochemical principles. Then, when a student moves from the classroom into the laboratory, all of the subtleties that modify the behavior of biomolecules, cells, and living organisms suddenly appear. Changes in pH, concentrations of ions, pressure, temperature, water activity, crowding from other macromolecules, and evolutionary constraints all complicate biomolecule structure and function.

Water stress experienced by cells—dehydration and drought, freezing and thawing, salt water or fresh water, and more—also cause physical stress on biomolecules. Each condition changes or impairs the function of proteins and nucleic acids, and requires cells to compensate. A common response to various water stresses throughout all domains of life is the accumulation of small organic molecules called osmolytes (Yancey & Siebenaller, 2015; Slama et al., 2015; Singh et al., 2022). Osmolytes help the cells retain water and also help maintain protein function. The ability of high concentrations of osmolytes (up to hundreds of millimolar) to support biomolecular function under environmental stress has led to their being called compatible solutes, while equally high concentrations of salts and many other solutes interfere with biomolecular function.

One function of osmolytes is to maintain protein folding under otherwise denaturing conditions. In the 1990s, Bolen and coworkers (Bolen & Baskakov, 2001) began using the phrase the “osmophobic effect” to describe this phenomenon. This name has the advantage of relating osmolyte effects to familiar hydrophobic effects in protein folding. Just as unfavorable interactions with water cause aggregation of hydrophobic surfaces, unfavorable interactions of molecules with osmolytes can favor their aggregation. In highlighting the analogy between osmolyte effects and the hydrophobic effect, the importance of water to osmolyte effects is emphasized, an important point in teaching deep, biochemical understanding. Unfortunately, the specific forces underlying the osmophobic effect are not as simple as the original presentation of the osmophobic effect implied.

#### Three elements: experiments, models, and communication

In this article, three interconnected elements are presented. The first is the work of over 20 undergraduate and high school students over several years. I had observed some significant quantitative disagreements between amino acid-osmolyte interactions determined by solubility measurements *versus* those determined by vapor pressure osmometry measurements. I selected glycine betaine (GB) interactions with glycine (Gly) and with glutamate (Glu) to attempt to understand and explain these differences. These were chosen because Gly-GB interactions, when corrected for solution non-ideality at high solute concentrations, agreed as measured by both methods (Cannon, Anderson & Record Jr, 2007), and Glu-GB interactions not only disagreed by a large amount quantitatively, but predicted qualitatively opposite effects (Capp et al., 2009; Auton & Bolen, 2007). This discrepancy has not been explicitly explained or corrected. My students learned how to accurately measure amino acid solubility in the presence of osmolytes and how to pass on their methods and results to students who followed them. The results presented here are those that multiple students were able to consistently reproduce. Part 1 presents these results and explains the difference between published solubility and osmometry measurements.

In part 2, I illustrate the computational and logical advantages of categorizing solvent accessible molecular surfaces by atom types over categorizing surfaces by groups like peptide backbone and amino acid side chains. I show that a model built on atomic surface types is computationally simpler than one built on functional groups, avoids pitfalls of the low resolution of a functional group model, and is generalizable beyond proteins. I review attempted applications of the transfer free energy and solute partitioning models which have been developed to predict protein-osmolyte interactions based on these two methods of accessible surface area (ASA) categorizations.

In part 3, I recommend a reframing of how we communicate the physical chemical ideas of osmolyte effects to make their understanding accessible to a broader audience while simultaneously focusing the field on developing the empirical model with the greatest potential for practical application. Both experimental and computational work since the formulation of the osmophobic effect support a larger role for the side chains than originally implied, and I briefly review those refinements. The “osmophobic effect” has largely fallen out of use in the literature, while researchers continue to discuss favorable and unfavorable interactions of osmolytes with different biomolecules or chemical groups. I propose reclaiming the phrase “osmophobic effect”, separating it from its ties to thinking about osmolyte effects as a majority protein backbone phenomenon, and adding the phrase “osmophilic effects” to expand the educational and descriptive advantages of our chosen vocabulary.

### Part 1. Reproducing Solubility Experiments

#### Methods

My students and I reproduced a subset of the experiments performed by Bolen and his collaborators determining the solubility of amino acids in the absence and presence of the osmolyte glycine betaine (GB) (Auton, 2004). We used the following chemicals: glycine (≥98.5%, Fisher Scientific, Fair Lawn, NJ, USA), monobasic sodium glutamate (L-glutamic acid monosodium salt monohydrate ≥98.0%, Aldrich, St. Louis, MO, USA), L-glutamic acid (99+%, Alfa Aesar, Heysham, England), sodium hydroxide (≥98%, Sigma Aldrich, St. Louis, MO), and betaine monohydrate (99+%, Acros Organics, New Jersey).

Solutions were prepared by weight to achieve the greatest precision. Volumes of solutions prepared were sufficiently large to assure a minimum of three significant figures of precision in all masses on an analytical balance sensitive to 10 µg, with a minimum final volume greater than six mL. Solutions were then incubated with regular mixing for 2–7 days near 25 °C, with final incubation before measurement for at least 2 h at 25–27 °C. I wish to note that we found these methods insufficiently accurate for low solubility amino acids, and I do not recommend them for low solubility compounds.

We measured glutamic acid density as a function of pH. Solutions were prepared by dissolving glutamic acid in volumes of 10.0 M sodium hydroxide, or 8.0 M sodium hydroxide and 1 M GB, estimated to achieve the desired pH. The solution was then titrated with the same or a diluted sodium hydroxide solution (still 1 M in GB when relevant) until the desired pH was obtained. The target volume was then completed by addition of water or 1 M GB. All concentrations were determined using the masses and densities of added components—never by volume. Final weights were determined after titration, and densities of sodium hydroxide and GB solutions used to calculate amounts of NaOH and GB added. For this manuscript I included only data between pH 6.0 and 7.99.

Solubility was determined from the intersection of two fitted lines: one following the increase in density as solute concentration increased, and a second fitting the densities of the saturated samples. The experimental solubility values sometimes varied by as much as 15% depending on whether the saturated solution fit was constrained to a horizontal line or allowed to slope. While these differences reflect imprecision in the method, they have no impact on the conclusions of this article. Because the solubility in the presence and absence of osmolyte shifted in the same direction, the solubility change was nearly identical by both fitting methods. Consequently I included values with the saturated data fit to sloping lines. This is explained further in Results.

I have also included in supplemental information (Fig. S1) a single experiment measuring the effects of GB on potassium glutamate solubility. These were done by my students at High Point University in 2011–12 using an incubator-shaker and an Anton-Paar density meter, by methods described previously (Liu & Bolen, 1995).

The current work, including earlier experiments as methods were refined, was performed over the course of approximately 10 years, entirely by high school and undergraduate students at Middle Georgia State University—most in their first or second year of college. Supplemental information includes graphs of data for glycine solubility measurements in GB (Fig. S2), as well as a spreadsheet including all fitted data. All students, including those who helped develop and refine the methods used, are included in the acknowledgments.

#### Results

My students reproduced a small selection of solubility measurements from a very large set of measurements (Auton, 2004). They measured interactions of glycine with glycine betaine (GB) to test the accuracy and precision of the methods, and simultaneously attempted to reproduce measurements of the interactions between glutamate and GB. Glutamate and GB were chosen because of the extreme difference between measurements of the their interactions using solubility measurements and using vapor pressure osmometry (VPO). I hoped to explain this difference. My students also made measurements with several nonpolar amino acids in water.

**Figure 1:**
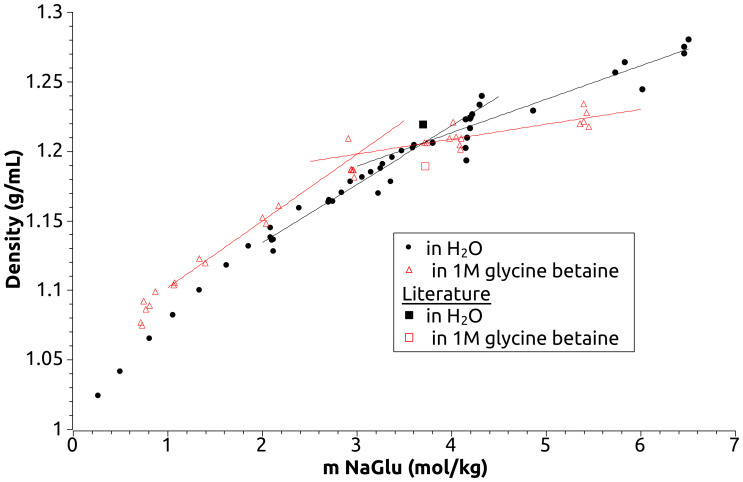
Sodium glutamate solubility in glycine betaine. Solubility of sodium glutamate, pH 6–8, in water (black circles) and in 1 M glycine betaine (red triangles). Squares represent solubility and density data from Auton, MT 2004 doctoral dissertation. Only these singular, tabulated values were provided by Auton. The concentration scale was converted from the original (g/100 g) using the reported 1 M GB solution density.

Several students independently measured the solubility of glycine in water and in 1 M GB to within 15% of reference values (shown in Supporting Information Fig. S2). Glutamate-GB solubility data were only included from students who accurately reproduced glycine-GB solubility data. Figure 1 shows the density of sodium glutamate solutions near pH 7 in the absence and presence of GB. The density increases as amounts of glutamate increase until an inflection point is reached where sodium glutamate is saturated. Students observed the presence or absence of solid in each sample, as well as measuring density. Our data do not have sufficient precision to fit a quadratic curve to unsaturated measurements, so we fit a line to solubility measurement data in water from 2–3.6 m NaGlu (unsaturated). We then fit data from 3.9–6.6 m NaGlu (saturated). For NaGlu in 1 M GB the solutions between 2.9–3.0 m NaGlu included both saturated and unsaturated samples (visual inspection), so we excluded these data from our fits. Reassuringly, the fitted lines intersect very near to three m. It is apparent in our reproduction that 1 M GB reduces the solubility of sodium glutamate by 0.6 m. This is a stark contrast with the slight increase in molal solubility reported previously (squares, Fig. 1) (Auton, 2004) and used in formulating the transfer model.

We calculated values for the change in chemical potential of glycine and glutamate as a function of GB molality, ${\mathrm{\mu }}_{23}^{ex}=\mathrm{\delta }{\mathrm{\mu }}_{2}^{ex}/\mathrm{\delta }{m}_{3}$ , where 3 is GB and 2 is glycine or glutamate, and the excess chemical potential is that due to specific interactions and not simply to mixing (see Supplemental Information for details). To compensate for the concentration differences between solubility and vapor pressure osmometry measurements we used the activity of single solute solutions to correct for solution non-ideality as done previously for analogous comparisons (Cannon, Anderson & Record Jr, 2007). These values are included in Table 1. Previous solubility studies did not report errors or provide raw data. Error calculations, after fitting the data as required to obtain these numbers, are highly ambiguous. I have not found a standard method for propagating error in fit coefficients that accurately reflects the scatter in the original data, so I have generously estimated the uncertainty as ±30%, reflecting the up to 15% error observed in each of our solubility measurements. The error is likely smaller, but the conclusions would remain the same with errors half or one and a half times as large.

**Table 1:** Comparison of preferential interactions (µ_23_/RT) determined by solubility and vapor pressure osmometry (VPO).

Uncorrected	Glycine-GB	Glutamate-GB
Previous solubility	0.21	−0.01
Current solubility	0.17 ± 0.05	0.21 ± 0.06
Non-ideality corrected		
Previous solubility	0.18 (Qu, Bolen & Bolen, 1998)	−0.01 (Auton, 2004)
Current solubility	0.15 ± 0.05	0.54 ± 0.18
Vapor pressure osmometry	0.15 ± 0.01 (Capp et al., 2009)	0.47 ± 0.01 (KGlu) (Capp et al., 2009)

**Notes.**

Concentration differences between solubility and VPO data corrected for non-ideality as described in Cannon, Anderson & Record Jr (2007). See Supplemental Information for calculations and further commentary.

In the first column of Table 1 (calculations provided in Supplemental Information), you can see that solubility measurements (Liu & Bolen, 1995; Qu, Bolen & Bolen, 1998) and osmometry (Capp et al., 2009) give similar values for interactions of glycine with GB, and that non-ideality corrections for glycine interactions with itself bring the values even closer to agreement. My students’ new measurements agree with both previously published results. In contrast, previously published solubility measurements for sodium glutamate (Auton, 2004) differ drastically from osmometric measurements of potassium glutamate (Capp et al., 2009). Correcting for non-ideality had no quantitative impact on the small interaction found by solubility measurements. The new solubility measurements depart markedly from those previously published, predicting sizable, unfavorable interactions. Although my students measured sodium glutamate instead of the potassium glutamate used in VPO measurements, our interaction value, corrected for concentration and non-ideality, agrees with the value determined by VPO. This is consistent with the finding that GB interacts primarily with glutamate and not the potassium ion, as shown by the small differences in GB interactions with NaCl and KCl (Capp et al., 2009). Further, a preliminary solubility study performed while working at High Point University gave results for KGlu in agreement with VPO measurements (Fig. S1), and was the impetus for these new solubility measurements.

#### Error in solubility measurements

My students and I identified a significant error in a solubility measurement that was used in developing the transfer model for predicting glycine betaine effects on protein folding (Auton & Bolen, 2007; Auton et al., 2011) (see Part 2). In addition, for high solubility molecules, corrections for non-ideality in amino acid self-interactions are required to obtain accurate values. Gathering the necessary data to make these corrections requires use of a method other than solubility measurements, like VPO or isopiestic distillation. These measurements can be used directly to determine amino acid-osmolyte interactions, and solubility measurements become redundant at best.

Other contradictions between solubility and VPO data have been previously reported (Cannon, Anderson & Record Jr, 2007; Guinn et al., 2011), and in Supplemental Information I provide a table (Table S1) of differences among published amino acid solubilities with differences up to 15%. My students’ new results support giving preference to VPO measurements and other measurements that are able to gather data closer to biological concentrations when building quantitative models of osmolyte effects. Solubility measurements need to be replicated or corroborated by closely related measurements, and where applicable, corrected for non-ideality effects.

This result is particularly significant because the transfer model developed by Bolen’s group (Auton et al., 2011) for GB-protein interactions, built solely on solubility data, predicted very small interactions between GB and anionic groups, while the solute partitioning model developed by Record’s group, built primarily on VPO data, predicted large, unfavorable interactions between GB and anionic groups. These differences lead to widely different predictions for interactions with protein surfaces rich in anionic groups. The results and observations presented here (Table S1) demonstrate that using unreplicated solubility measurements as the basis of a predictive empirical model has a significant chance of inaccuracies. In the case of GB, solubility model data poorly predicted GB effects on protein stability, with one of the three test cases predicting the wrong sign for the m-value, and the other two only having moderate accuracy (supporting information in Auton et al. (2011). GB is apparently the solute with the poorest predictions in the study, but most of the osmolyte predictions included cases with significant differences from experimental m-values. Replicated, high precision solubility measurements for compounds with low solubility could still be useful as quantitative inputs to empirical models (Guinn et al., 2011; Diehl et al., 2013), but the large, unreplicated data set used by Auton et al. needs to be used with caution.

I would predict that additional replications of earlier solubility experiments would reveal few significant errors, however, in Part 2 I will explore why the side chain/backbone division of protein surfaces, which has historically been tied to solubility studies, is not an optimal choice for quantifying osmolyte effects, and why an atomic surface type based model, associated with osmometry (and selectively incorporated solubility data and data from other methods), is a stronger candidate for a useful, predictive model of osmolyte effects.

### Part 2. Atom Type Model *vs.* Functional Group Model for Predicting Osmolyte-Biopolymer Interactions

#### Predictive and interpretive models of osmolyte effects

Numerous models have been used to explore and explain osmolyte effects in different contexts. Models variously apply to individual osmolytes (Capp et al., 2009; Diehl et al., 2013; Bhojane et al., 2016; Guinn et al., 2013; Cheng et al., 2017; Hong, Gierasch & Liu, 2015; Knowles et al., 2015; Cheng et al., 2016; Moeser & Horinek, 2014), attempt to characterize atomic level mechanisms (Hu et al., 2010; Canchi et al., 2012; Ganguly et al., 2022), employ challenging thermodynamic or quantum mechanical calculations (Ma et al., 2010; Shkel & Record Jr, 2004), utilize computational software requiring significant training and/or computational power (Moeser & Horinek, 2014; Ganguly et al., 2022; Canchi & García, 2013; Tomar, Asthagiri & Weber, 2013), or otherwise require specialization. These conditions limit application to narrow groups of researchers. Two models for predicting and interpreting osmolyte effects have been put forward that only require knowledge and computing resources available to most biochemical researchers. One is based on the transfer free energy model for urea denaturing effects proposed by Tanford (1970). Tanford’s model divided proteins up into peptide backbone and side chain functional groups. He measured the solubility of amino acids in water and in urea solutions. The change in solubility gave a quantitative measure of how urea, the amino acid, and water all interact with each other. In the 1990s and 2000s, Bolen and his collaborators extended this model to a variety of osmolytes in addition to urea, and provided methods that would allow other researchers to calculate osmolyte mediated stabilization or destabilization of proteins in many contexts (Auton & Bolen, 2007). This model was applied to an impressive array of protein thermal stability data in 2011 (Auton et al., 2011). Experiment and prediction agreed in many cases, but with some notable exceptions. I will refer to this model as a functional group-based model.

At the same time as Tanford’s (1970) functional group-based model was being expanded, another model, also inspired by the ideas of Tanford and Timasheff, was proposed by Record & Anderson (1995). This model added the idea of osmolytes partitioning between a local water domain near biopolymer surface and a bulk domain one or two water molecules distant from the surface, bringing water more explicitly into the model. Record and colleagues chose an atom-based characterization of protein surface (Felitsky & Record Jr, 2004) rather than the side chain/backbone based characterization of the functional group-based model. This atom-based model relied on the idea that osmolytes and biopolymer surfaces interact preferentially with one another or with water. I will refer to atom-based preferential interaction models as osmophobic/osmophilic (OO) models for reasons I will expand on in Part 3. Despite the theoretical underpinning of the solute partitioning model having an additional level of complexity compared with the transfer free energy model, application of an atom-based OO model to predicting and interpreting osmolyte effects requires nearly identical skills and computational power as application of the transfer model.

In this part I will illustrate the advantages of modeling proteins by atomic surface type over functional group surfaces, and explain the greater simplicity and generalizability of an atomic surface type osmophobic/osmophilic model.

##### Method note

The unfolded Trp-cage protein model was built using PyMOL’s build tool with an extended β-sheet conformation. Images of proteins were also generated using PyMOL. (The PyMOL Molecular Graphics System, Version 2.3.0 Schrödinger, LLC.) Accessible surface areas were calculated using the GETAREA algorithm developed by Fraczkiewicz & Braun (1998). The coordinate file for the extended chain is included in Supplemental Information.

#### Illustrated comparison of predictive osmolyte models

##### Groups *vs.* atoms

Two natural ways to model interactions between biopolymers and the many molecules that surround them are to (1) divide the biopolymers into molecular groups—like the protein backbone and amino acid side chains, or the sugar-phosphate backbone of nucleic acids and the aromatic nucleobases, or polar lipid head groups and nonpolar tails—or (2) into smaller, atomic surface categories—like anionic oxygen, cationic nitrogen, aromatic carbon, *etc*. It is beyond the scope of this article to explore the many thoughtful motivations for these different choices, so I focus only on the consequences for measuring and predicting osmolyte effects on biopolymers.

**Figure 2:**
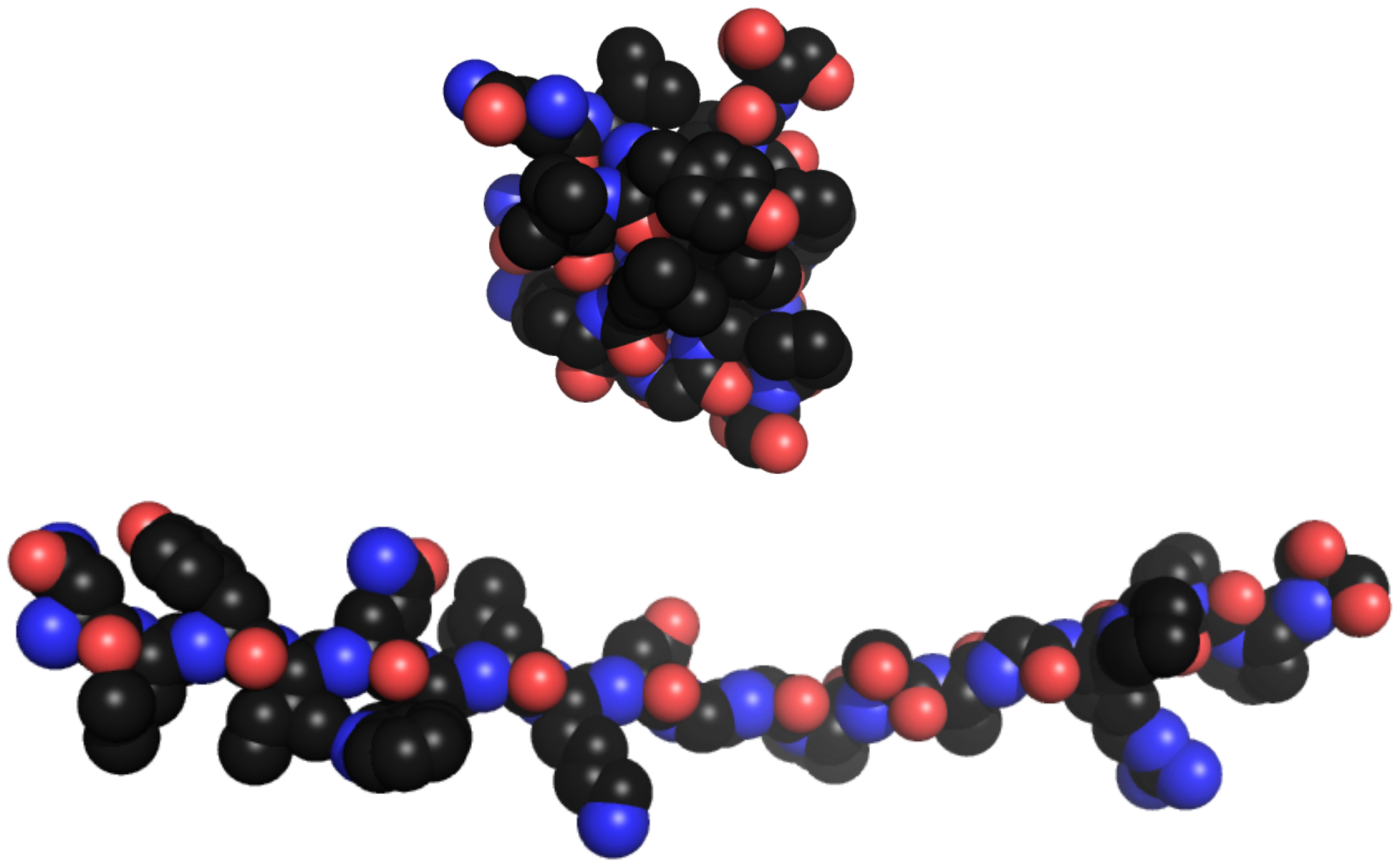
Trp-cage protein structures. Trp-cage protein in folded (top, 1L2B. pdb) and extended β-strand (bottom, constructed with PyMOL) conformations shown without hydrogen atoms. Hydrogen atoms are not included in structures when calculating accessible surface areas.

To illustrate the differences, I will use a structure of the Trp-cage protein (Fig. 2), and a model of the Trp-cage protein as an extended β-chain (a simple model of the unfolded state often used in calculating osmolyte effects on protein stability). Using GetArea, a program that rolls an imaginary water molecule around the structures and reports water accessible surface area (ASA) for the biopolymer, we can obtain ASA for every atom, backbone segment, and side chain. By taking the differences between the extended and folded structures we can calculate the change in ASA when the protein unfolds (ΔASA). Table 2 summarizes all of these different surface categories for the Trp-cage peptide. Further notes and a small example for calculating ΔASA of specific residues are included in Supplemental Information.

**Table 2:** Accessible surface areas (in Å^2^) for folded and extended conformations of the Trp-cage protein.

**OO Atom-based model**				**Functional group-based model**			
Surface type	Folded ASA	Extended ASA	ΔASA	Surface type	Folded ASA	Extended ASA	ΔASA
amide C-sp2	36.28	23.06	−13.22	backbone	433.03	795.15	362.12
aliphatic C-sp3	1,100.67	1,602.87	502.2	Arg	99.61	189.38	89.77
aromatic C-sp2	66.58	270.52	203.94	Asn	124.39	102.03	−22.36
carboxylic C-sp2	2.19	1.81	−0.38	Asp	34.8	112.69	77.89
amide N-sp2	105.44	152.59	47.15	Gln	106.69	116.85	10.16
cationic N-sp3	124.98	174.13	49.15	Gly	0	0	0
aromatic N-sp2	0	4.41	4.41	Ile	118.71	108.92	−9.79
amide O-sp2	216.29	439.11	222.82	Leu	191.75	274.56	82.81
anionic O-sp2	103.89	154.92	51.03	Lys	102.53	169.1	66.57
hydroxyl O-sp3	93.96	129.75	35.79	Pro	294.14	433.65	139.51
				Ser	198.32	242.34	44.02
				Trp	17.95	197.03	179.08
				Tyr	93.57	169.34	75.77
Additional surfaces required to extend each model to all proteins and nucleic acids							
few additional atom types	*e.g.*, S-sp3			additional side chains	*e.g.*, 8+ remaining amino acids side chains		
for nucleic acids	*e.g.*, Phosphate O, neutral N-sp3, possibly acetal O-sp3			nucleic acid functional groups	*e.g.*, phosphate, sugars, 5+ nucleobases		
Total	∼4 additional atom types			Total	∼15+ groups		

**Notes.**

ASA calculations performed using GETAREA (Fraczkiewicz & Braun, 1998) with Trp-cage structures from 1L2Y.pdb and an extended β-strand structure built with PyMOL. Coordinates for the extended strand are included in Supplemental Information.

##### Computational complexity

At a glance, the number of surface categories is similar for constructing both the osmophobic/osmophilic and transfer models: 10 types of atomic surface compared with 12 side chains and the peptide backbone. As one moves to more complex proteins, however, both the number of surfaces categories required to perform calculations increases much faster for the transfer model than for an atom-based OO model. The categories of atoms (*e.g.*, C, N, O, P with sp, sp^2^, sp^3^ hybridization, *etc.*) are very limited for organic molecules, while the diversity of side chains and other functional groups used to categorize biomolecules is much larger. For an OO model, osmolyte interactions with virtually any model compound containing the atomic surfaces of interest can be used to generate data for the model. This includes nearly all organic molecules. Predictions of osmolyte effects can also be made for uncommon or synthetic amino acids, as long as they contain these same atom types. For the transfer model, osmolyte interactions need to be measured with every side chain of interest, and there is no natural way to combine data from one side chain to another, or expand the predictions to other biomolecules (like nucleic acids) without collecting data for all the additional functional groups.

The bottom part of Table 2 indicates how many additional surface groups need to be included to extend functional group-based models and atom-based models to not only other proteins, but also to nucleic acids and protein-nucleic acid interactions. Functional group-based models require every amino acid side chain, the sugar-phosphate backbone, every nucleoside found in the nucleic acid, and any uncommon side chains or nucleosides. Every extension of functional group-based models to new biopolymers, or to other molecules like protein ligands, requires new sets of model data. To just cover basic proteins and nucleic acids would require 28+ additional surface categories. An osmophobic/osmophilic model can use values for surface categories from any model compounds to build and refine its predictive capacity. Most nucleoside and sugar surfaces, and many other biological molecules, already have estimates that can be derived from the amino acid-osmolyte interaction data. Only four additional surface categories, like phosphate oxygen surface, are required to extend the model to osmolyte-nucleic acid interactions. Further, any measurements of osmolyte-nucleic acid interactions can be used to refine the values for each of the atomic surface categories already included in the model. Thus, with approximately 12 atom categories, an OO model can make predictions for proteins, nucleic acids, carbohydrates, and potentially a wide array of organic molecules and structures as long as ASA can be determined. It can also be constructed and refined with inputs from all of these sources.

##### Predictive capacity

Another difference between the osmophobic/osmophilic atom-based models and functional group-based models is one of resolution. To illustrate this I will highlight the two serine residues at positions 13 and 14 in the Trp-cage protein, and compare them with the fully exposed serine side chain ASA used for building functional group-based models (sample ASA calculations included in Supplemental Information). As the Trp-cage protein unfolds, only 11 Å^2^ of ASA are exposed on Ser-13, while 75 Å^2^ are exposed of Ser-14. When a serine molecule is transferred from water into an osmolyte solution, approximately 84 Å^2^ of side chain surface are exposed to water. More significantly, the 84 Å^2^ exposed to water are approximately 38% O and 62% C. Ser-13 exposes 11 Å^2^ of O, while Ser-14 exposes only approximately twice that (23 Å^2^) and the transferred side chain three times that (32 Å^2^). The amount of C surface exposed or transferred changes more dramatically, from 0 Å^2^ for Ser-13, to 23 Å^2^ for Ser-14, to 52 Å^2^ for an exposed Ser side chain. The percentage changes are listed in Table 3. So functional group-based models are built on compounds with a ratio of 62:38 carbon to oxygen, while residues in proteins may expose vastly different ratios of carbon and oxygen to the solvent. As noted, neither Ser-13, nor Ser-14 matches this ratio.

**Table 3:** Changes in ASA (Å^2^) for serine side chains between the folded and extended states of the Trp-cage protein and for transfer of a serine side chain from water to osmolyte solution.

	ΔASA	%ΔASA-O	%ΔASA-C
Ser-13	10.56	100	0
Ser-14	74.47	31	69
Fully exposed side chain	84	38	62

#### Atom-based models preferable for empirical prediction

The observations I have made about accessible surface area categorization have been available behind all of the previous reports and comparisons of Bolen’s transfer model and Record’s solute partitioning model, but I have not found any reports where the significance of the differences has been made bluntly clear (Auton & Bolen, 2007; Auton et al., 2011). The ASAs of fully exposed side chains have different compositions from side chains exposed in either native proteins or protein unfolding. This is unavoidably true for all heteroatomic side chains. Building a model based on the ASA of functional groups, while using fully exposed functional groups to represent partially exposed functional groups found in real proteins and protein processes, will be flawed. There is no way around the ASA composition discrepancy while retaining the side chain/backbone breakdown of protein surfaces. One can only hope that differences average out over a large number of groups, or that they don’t matter, or that the particular details of the breakdown fortuitously fix any problems.

Computational models could be an alternative for predicting osmolyte effects without the ASA composition discrepancy that functional group-based models introduce. But all atom computational modeling requires specialization and large amounts of computing power, and simplified computational models will require forcefields that do not currently exist, built on experimental osmolyte effect data.

In contrast, atom-based models, while perhaps not capturing the detail of all atom computational models, avoid the composition discrepancies of functional group-based models by characterizing both experimental inputs and predictive applications in terms of atomic surface categories. An atom-based model can capture the ASA differences between two amino acids of the same type in two different molecular environments—something functional group-based models necessarily fail to do. Further, atom-based models can be extended more easily to a larger variety of biological molecules, with about a dozen categories as opposed to three dozen or more for functional group-based models.

In summary, an osmophobic/osmophilic model (1) is computationally smaller than a functional group-based model, (2) is more easily extended to all proteins and other biomolecules (including uncommon and synthetic amino acids, nucleic acids, carbohydrates, *etc.*), (3) avoids issues of resolution that arise from treating an entire side chain as a single surface type, (4) can be built through experiments employing a wider array of model compounds, and (5) can make predictions about osmolyte effects on many organic molecules and molecular interactions. While all of these observations have been implicit in previously published work, I have not found an explicit statement or illustration of these reasons for preferring an atom-based model.

In an independent test of Bolen’s functional group-based transfer model (Auton et al., 2011) and Record’s atom-based solute partitioning model (Guinn et al., 2011) Silvers & Myers (2013) compared the predictive accuracy of the transfer and OO models. For unfolding one particular protein, as of 2013 formulations, the functional group-based model predicted osmolyte effects for nine osmolytes with errors of ∼70–150% with an average error near 100%. The atom-based model only made predictions for three osmolytes, but with 2–6 times greater accuracy than the transfer model with errors of ∼25, 40, and 70%. While there is clearly room for improvement of the solute partitioning model, extending and refining this model promises more broadly useful and accurate quantitative predictions of osmolyte effects.

## Part 3. Communicating Physical Chemistry of Osmolytes to Many Fields

The physical chemistry of osmolytes is a small field, but one with broad interest. Interest continues in understanding osmolyte effects on protein stability, not only within physical chemistry, like in characterizing biochemical intermediates (Motlagh et al., 2016; Hong et al., 2005; Gries et al., 2010; Sengupta et al., 2017), but also osmolytes’ uses in stabilizing marketable protein products like drugs(Sudrik et al., 2019; Nelson et al., 2012), or synthetic spider silk (McLachlan, Gandjian & Alhumaidan, 2016). Protein aggregation and amyloid formation (Sukenik & Harries, 2012; Sukenik et al., 2011; Gao & Winter, 2015; Macchi et al., 2012; Aravindan et al., 2020; Rabbani & Choi, 2018) is another area where the effects of osmolytes are noticed but incompletely understood. Molecular crowding affects these processes, but studies have repeatedly found that osmolyte specific effects contribute measureably (Sukenik et al., 2011; Gorensek-Benitez et al., 2017). Computer models of biopolymer behavior in increasingly complex environments (for example, a cell) require both data and quantitative models of osmolyte effects upon which to build efficient and accurate algorithms (Ma et al., 2010; Canchi & García, 2013; Jahan & Nayeem, 2019). Osmolytes are important in botany, particularly in understanding plant responses to salt stress (Slama et al., 2015; Singh et al., 2022), and in marine biology in understanding abiotic stresses on proteins (Somero, 2022). Osmolytes and osmosensing are crucial to bacterial survival (Wood et al., 2001; Rahman et al., 2018). This is just a sampling of research areas involving osmolytes.

It is possible that better, unifying language that simplifies transfer of ideas across physical chemical disciplines and into biochemistry and biology could help advance research and understanding in these disparate fields. Such language needs to be simple, memorable, and convey accurate information. The “osmophobic effect” accomplished the first two admirably. The parallels with the hydrophobic effect made it memorable, and the idea that osmolytes stabilized proteins primarily through unfavorable interactions with the peptide backbone was simple. In Parts 1 and 2 we saw that this idea is quantitatively inaccurate and was even revised by its original formulators to acknowledge significant side chain contributions by 2011 (Auton et al., 2011). Yet despite the inaccuracy of the original “osmophobic effect”, and the availability of an equally simple and more accurate model, the idea of the osmophobic effect has persisted (Sharma et al., 2021; Canepa et al., 2020; Pepelnjak et al., 2024).

Osmolytes vary in how they influence proteins, not always scaling with peptide backbone exposure. Some studies indicate favorable interactions between biopolymers and some osmolytes, implying interactions better described as “osmophilic” effects (Diehl et al., 2013; Bhojane et al., 2016; Acharyya et al., 2020; Duff & Howell, 2015). Numerous studies reveal the need for including protein side chain contributions in interpreting osmolyte effects (Capp et al., 2009; Auton et al., 2011; Moeser & Horinek, 2014; Canepa et al., 2020; Pepelnjak et al., 2024; Acharyya et al., 2020; Stanley & Rau, 2008; Duff & Howell, 2015; Stanley & Rau, 2008; Holehouse et al., 2015; Murdock et al., 2014; Olgenblum, Carmon & Harries, 2023; Stasiulewicz et al., 2022). The osmophobic effect fails to address interactions with nucleic acids, where additional osmophilic effects have been observed (Lambert & Draper, 2007; Schwinefus et al., 2013). The simple osmophobic effect also fails in application to native proteins (Felitsky et al., 2004) and intrinsically disordered proteins (Pepelnjak et al., 2024). Despite these technical failings, it may be valuable to reclaim the language of the osmophobic effect. Osmophobic and osmophilic effects, associated with more accurate and useful quantitative models, could help communicate physical chemical ideas simply, memorably, and accurately across a breadth of interested disciplines.

## Conclusions

### Reframing osmophobic and osmophilic effects

Osmolytes do not repel the peptide backbone—or at least the picture is not that simple. Every small, compatible, organic molecule interacts with proteins—and with nucleic acids, and certainly with carbohydrates and lipid assemblies as well. Yet the power and simplicity of that “osmophobic effect” idea and language took hold with such strength that, despite its abandonment (in its simplest form) by its original proponents, and the many studies that have disproved the simplicity and provided alternative, potentially more useful and accurate models. Despite experts in the thermodynamics of osmolyte effects having ceased to use the term osmophobic effect in association this simplistic view, the idea persists on the periphery that osmolytes repel the peptide backbone, and that is why they work (Sharma et al., 2021; Canepa et al., 2020; Pepelnjak et al., 2024).

There are simple, quantitative and qualitative ways to talk about osmolyte interactions with biopolymers. Just as Tanford found that urea-protein interactions could be usefully approximated as an attraction of the peptide backbone for urea, GB interactions with proteins and nucleic acids can be explained primarily as unfavorable interactions with anionic oxygen (Felitsky & Record Jr, 2004; Felitsky et al., 2004). But with a little more work it is computationally and visually simple to show exclusions of GB from oxygen, anionic oxygen, and aliphatic carbon surfaces and also noticeable attraction to nitrogen, cationic nitrogen, and aromatic surfaces (Diehl et al., 2013; Bhojane et al., 2016). For an intuitive example of this see Figure 5 of Diehl et al. (2013). Extension of the osmophobic/osmophilic solute partitioning model to other osmolytes can provide similar, simple ways to talk about attractions and repulsions, or accumulation and exclusion, of osmolytes at biopolymer surfaces. Osmophilic attractions will destabilize structures, and osmophobic repulsions will stabilize structures or complexes. Osmophobic repulsions will help cells retain water and hydrate biopolymers under water stress. Osmophilic attractions will do the opposite. Researchers can apply the simple tool of examining atomic surfaces to make qualitative and quantitative predictions about the effects of a particular osmolyte on the structure of interest.

This is a particularly attractive framing because so many people who have had even one semester of biochemistry will have encountered the hydrophobic effect. They will understand at a basic level that non-polar atomic surfaces cluster together in water because they are hydrophobic, and that this clustering stabilizes proteins and membranes. Teaching them that osmophobic surfaces will hide away from an osmolyte, thus stabilizing a structure, and that osmophilic surfaces will be attracted to solvent, destabilizing a structure, is a small, memorable, accurate, and natural extension of a concept they are already familiar with.

In addition, the tools necessary for application of the osmophobic/osmophilic solute partitioning model are accessible both economically (free software) and educationally. All of the programs can be used by working through brief tutorials available with a web search. These features create the potential to take osmolytes beyond being a biologically omnipresent sideshow, treated with qualitative uncertainty, and turn them into a quantitative tool upon which hypotheses can be formulated, and data can be analyzed. While physical chemists researching osmolytes will need to continue to refine models and explore and argue over important details of method and theory, we can simultaneously encourage researchers in other areas to contribute through straightforward application of simple, predictive, quantitative models. Osmolytes could become a tool used to understand a wide array of biochemical problems. We can turn from asking, “how do osmolytes work?” to asking specific questions like, “can I use this osmolyte to quantify structural changes in an intrinsically disordered protein when it binds a ligand?” “Can I explain why this osmolyte interferes with one particular protein and not another?” Biochemists, botanists, and microbiologists could shift from qualitative pronouncements about the effects of osmolytes to recognizing osmophobic and osmophilic effects as quantitatively useful tools in their toolbox.

## Supplemental Information

10.7717/peerj.20623/supp-1 Supplemental Information 1 Raw solubility data for glycine and sodium glutamate in water and 1 M glycine betaine solution Solubility Data used in the manuscript. Data included are molal concentrations of glycine or glutamate, density, and pH of each sample in either water or 1 M glycine betaine. Each data point is a single measurement. Graphical representations of the data are in the manuscript. Data from training experiments (first attempts and unreproduced attempts), and method development (comparison of specific gravity bottle, micropipette, and volumetric pipette for measuring volume) were not included. Most student work from 2012-2017 was on method development and learning to reproduce work over constantly changing student researchers, and their results were not included in the manuscript or supplemental data.

10.7717/peerj.20623/supp-2 Supplemental Information 2 Solubility of potassium glutamate in water and in 1 M glycine betaine Potassium glutamate solubility in water and in 1 M glycine betaine solution. Data collected in 2011-2012 at High Point University. The small arrows represent reported solubility of sodium glutamate in water and 1 M glycine betaine Auton, 2004 dissertation. Complete data sets were never published). This was our first indication that there might have been flaws in some reported solubility data. Raw data are no longer available. Solubility values for monopotassium glutamate are not on PubChem, and I have not succeeded in finding published values.

10.7717/peerj.20623/supp-3 Supplemental Information 3 Solubility of glyine in water and in 1 M glycine betaine Glycine solubility in water (black circles) and in 1 M glycine betaine (red triangles). Our solubility determinations were both lower than reported values, but within 10% (in water, black filled square, Qu et al., 1998; in 1M GB, blue open square, Auton, 2004 dissertation. Complete data sets were never published). Constraining the saturated solution fit to a horizontal line results in solubility values closer to the previously reported solubilities, but for consistency of analysis we allowed the saturated solution fit to slope. Our change in solubility values from water to 1 M GB were nearly identical with either fitting method.

10.7717/peerj.20623/supp-4 Supplemental Information 4 Explanation of calculations and published discrepancies in solubility data Equations used for calculating preferential interactions between solutes and osmolytes, including how to correct for non-ideality, and data sources used for those corrections.
A table comparing published values for amino acid solubility from different sources, and a brief discussion of the differences and their implications.
A description of the steps used to determine and calculate changes in accessible surface areas of the Trp-cage protein and its residues

10.7717/peerj.20623/supp-5 Supplemental Information 5 Calculations for preferential interactions from solubility data and changes in Serine residue accessible surface areas The first spreadsheet shows the specific values and calculations to obtain the numbers in Table 1, preferential interactions of glycine and glutamate with glycine betaine, from solubility measurements. The second sheet shows the results from GetArea and the calculations for changes in water accessible surface area for serine residues 13 and 14 in the Trp-cage protein. The equations and data sources are explained in the main text and the Supplemental Information text document.

10.7717/peerj.20623/supp-6 Supplemental Information 6 PDB coordinates for extended Beta-strand Trp-cage protein The pdb coordinates generated from the PyMol extended Beta-strand structure of the Trp-cage protein corresponding to pdb entry 1L2Y. These coordinates were submitted to GetArea to obtain water accessible surface areas for the unfolded protein.

## Additional Information and Declarations

### Data Availability

The following information was supplied regarding data availability: The raw data is available in the Supplemental File.
