# Supplementary figures and images for: Osmolyte effects: revisiting solubility measurements, accessible surface area categorization, and language for communicating with a broad audience
Source: PeerJ. 2026 Mar 27;14:e20623. doi: 10.7717/peerj.20623 (PMC13034864; doi:10.7717/peerj.20623)

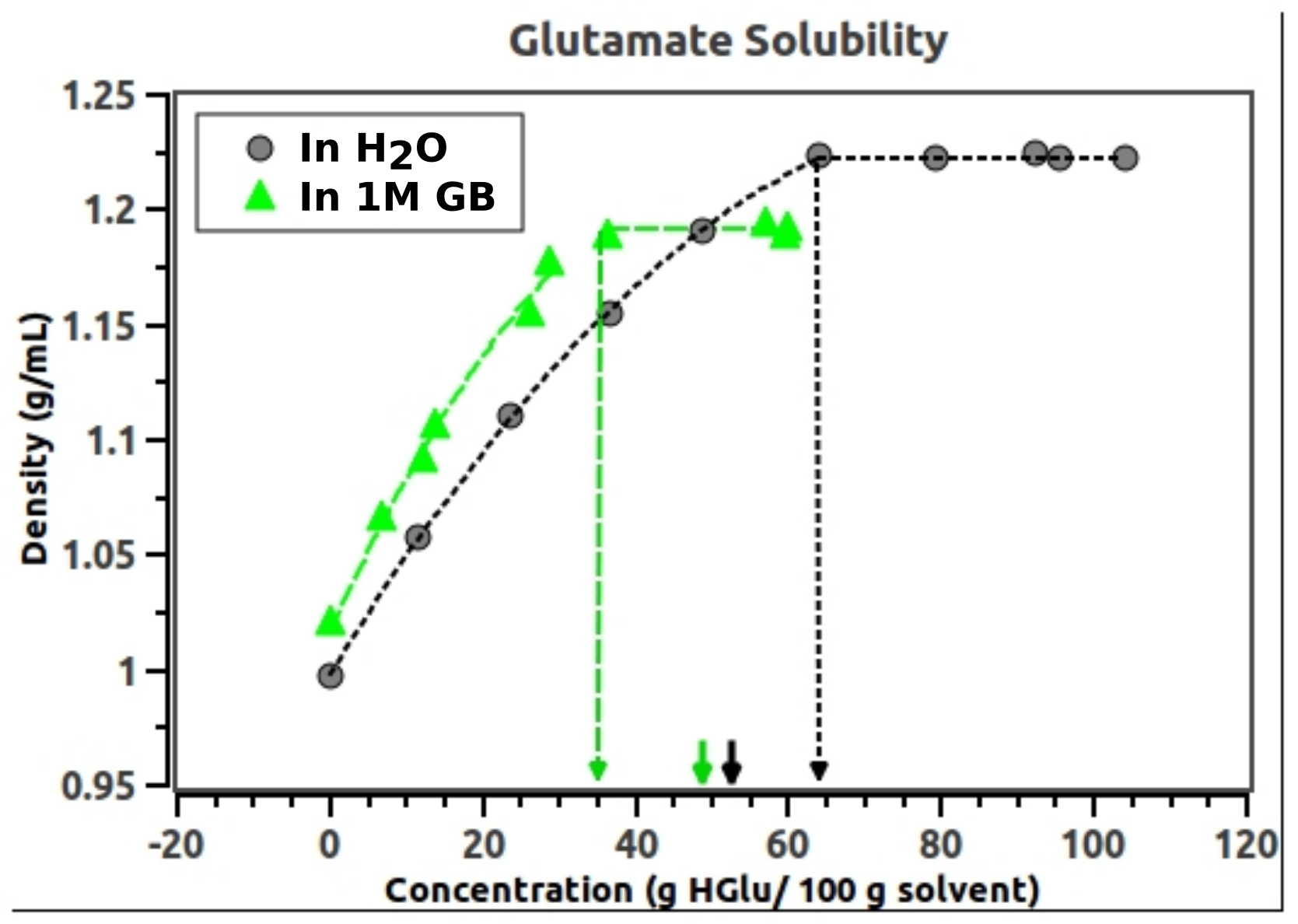

Supplement: Supplemental Information 2 — Potassium glutamate solubility in water and in 1 M glycine betaine solution. Data collected in 2011-2012 at High Point University. The small arrows represent reported solubility of sodium glutamate in water and 1 M glycine betaine Auton, 2004 dissertation. Complete data sets were never published). This was our first indication that there might have been flaws in some reported solubility data. Raw data are no longer available. Solubility values for monopotassium glutamate are not on PubChem, and I have not succeeded in finding published values. [file peerj-14-20623-s002.png]

# Glycine Solubility

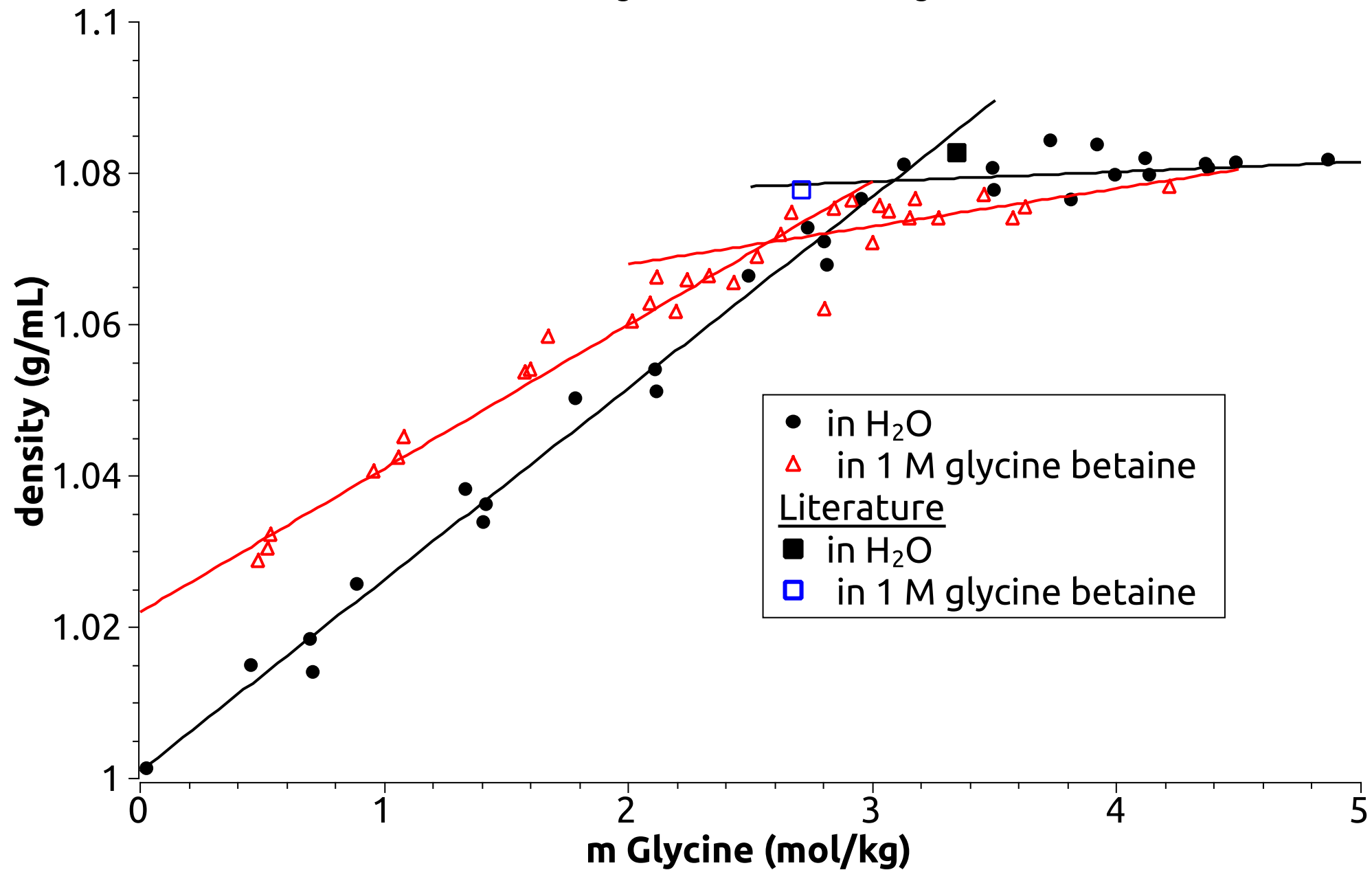

Supplement: Supplemental Information 3 — Glycine solubility in water (black circles) and in 1 M glycine betaine (red triangles). Our solubility determinations were both lower than reported values, but within 10% (in water, black filled square, Qu et al., 1998; in 1M GB, blue open square, Auton, 2004 dissertation. Complete data sets were never published). Constraining the saturated solution fit to a horizontal line results in solubility values closer to the previously reported solubilities, but for consistency of analysis we allowed the saturated solution fit to slope. Our change in solubility values from water to 1 M GB were nearly identical with either fitting method. [file peerj-14-20623-s003.pdf]
