# Supplementary material for: Osmolyte effects: revisiting solubility measurements, accessible surface area categorization, and language for communicating with a broad audience
Source: PeerJ. 2026 Mar 27;14:e20623. doi: 10.7717/peerj.20623 (PMC13034864; doi:10.7717/peerj.20623)
Supplement: Supplemental Information 4 — Equations used for calculating preferential interactions between solutes and osmolytes, including how to correct for non-ideality, and data sources used for those corrections. A table comparing published values for amino acid solubility from different sources, and a brief discussion of the differences and their implications. A description of the steps used to determine and calculate changes in accessible surface areas of the Trp-cage protein and its residues [file peerj-14-20623-s004.docx]

# Supplemental Information for "Reframing Osmophobic and Osmophilic Effects"

# Correcting for non-ideality

### Calculation of preferential interactions:

There are different ways to represent interactions between solute molecules. One that is relatively easy to compare among different methods (among other advantages) is the change in excess chemical potential (not due to mixing) of one solute (component 2, the amino acid) as a function of the concentration of the other solute (component 3, the osmolyte). In brief:


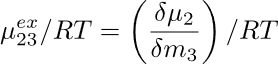
 (Equation 1)

As the concentration of 2 approaches zero (e.g. low solubility), this can be calculated as:


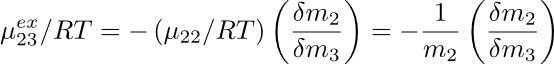
 (Equation 2)

When m_2_ is not small,
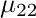
 must be corrected for non-ideality of 2. This can be approximated by fitting water activity data (component 1) for a solution of solute 2 to obtain a value for
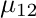
 at the desired
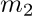
. The non-ideality corrected value is then:


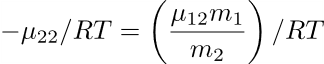
 where
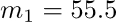
 (Equation 3)

I used the same data sources and non-ideality corrections as previously published (glycine: Ellerton, H. D.; Reinfelds, G.; Mulcahy, D. E.; Dunlop, P. J. *J. Phys. Chem.* 1964, 68, 398. as reported in Cannon, Anderson, and Record 2007; sodium glutamate: Oscar D. Bonner *J. Chem. Eng. Data* 1981, 26, 2, 147–148).

### Discrepancies in solubility data from different sources

For low solubility compounds, non-ideality corrections are not significant in calculating preferential interactions. For high solubility compounds such corrections require extensive activity data for each solute. These activity data are only available for glycine and a small selection of osmolytes and other biological molecules. For most of these molecules which have sufficient activity data over a wide concentration range, including glycine betaine, the data were obtained by vapor pressure osmometry. Osmometry can be and was used to directly determine preferential interactions at more biologically relevant concentrations, eliminating the need for solubility measurements.

Reported solubilities of amino acids in water have shown varied reproducibility. Table S1 below compares values reported by Bolen’s research group with those reported by Nozaki and Tanford, and with the 2003 Handbook of Aqueous Solubility Data as reported on PubChem.ncbi.nlm.nih.gov. As is evident in the table, solubility in water may have errors as large as 10-15%, and errors in the presence of osmolytes are largely unknown, as very few experiments have been reproduced.

| Supplemental Table S1. Comparison of solubility of amino acids from different published sources. | | | | | | | |
| --- | --- | --- | --- | --- | --- | --- | --- |
|  | Data Sources | | | | | | |
|  | Liu^a^ | Qu^b^ | Nozaki^c^ | Handbook^d^ | Liu^a^ | Qu^b^ | Nozaki^c^ |
| Amino Acid^e^ | Solubility(g solute/100 g H_2_O) | | | | % difference from Handbook of Aqueous Solubility Data 2003 | | |
| Met | 5.75 | 5.69 | 5.59 | 5.66 | 1.6 | 0.5 | -1.2 |
| Leu | 2.16 | 2.15 | 2.16 | 2.15 | 0.5 | 0.0 | 0.5 |
| Thr | 9.73 | 9.74 | 9.8 | 9.7 | 0.3 | 0.4 | 1.0 |
| Gly | 25.1 | 25.1 | 25.1 | 24.9 | 0.8 | 0.8 | 0.8 |
| Ser | 42.9 | 42.9 |  | 42.5 | 0.9 | 0.9 |  |
| Trp | 1.33 | 1.36 | 1.38 | 1.34 | -0.7 | 1.5 | 3.0 |
| Ala | 16.6 | 16.6 | 16.7 | 16.4 | 1.2 | 1.2 | 1.8 |
| Gln | 4.08 | 4.19 | 4.3 | 4.13 | -1.2 | 1.5 | 4.1 |
| Val | 5.73 | 5.73 |  | 5.85 | -2.1 | -2.1 |  |
| Ile | 3.35 | 3.35 |  | 3.44 | -2.6 | -2.6 |  |
| Tyr | 0.0469 | 0.0469 | 0.0451 | 0.0479 | -2.1 | -2.1 | -5.8 |
| His | 4.3 | 4.3 | 4.33 | 4.56 | -5.7 | -5.7 | -5.0 |
| Phe | 2.82 | 2.81 | 2.8 | 2.64 | 6.8 | 6.4 | 6.1 |
| Asn | 2.76 | 2.64 | 2.51 | 2.94 | -6.1 | -10.2 | -14.6 |
| Pro | 181.5 | 181.5 |  | 162 | 12.0 | 12.0 |  |
| NaGlu | 62.4 | 62.4 |  | 73.9 | -15.6 | -15.6 |  |
| LysHCl | 71.3 | 71.3 |  |  |  |  |  |
| NaAsp | 77.9 | 77.9 |  |  |  |  |  |
| ArgHCl | 85.3 | 85.3 |  |  |  |  |  |
| ^a^ Liu, Y.; Bolen, D. W., *Biochemistry* 1995, 34 (39), 12884–12891.  ^b^ Qu, Y.; Bolen, C. L.; Bolen, D. W., *Proc. Natl. Acad. Sci. U. S. A.* 1998, 95 (16), 9268–9273.  ^c^ Nozaki, Y.; Tanford, C., *J Biol Chem* 1963, 238, 4074–4081.  ^d^ The Handbook of Aqueous Solubility Data 2003, accessed from PubChem.ncbi.nlm.nih.gov entries for each amino acid.  ^e^ Amino acids are ordered from smallest differences between the Handbook of Aqueous Solubility Data and the other data to largest differences. | | | | | | | |

One might think that 10-15% maximum error, with a median error of 1.8%, might not be a big problem. It is important to remember that osmolyte-amino acid interactions are calculated from the differences between similar size numbers. The average change in solubility of amino acids in the presence of 1 M glycine betaine (Matthew Auton thesis, comparison not shown) is about 14%. Two compounding errors of 1.4% in opposite directions will result in a 20% systematic error in calculating interactions. A combined 15% error in measuring solubility in the presence and absence of osmolyte could result in predicting the opposite direction of interaction between amino acid and osmolyte. From this table we know that differences as large as 15% are possible in published solubility data, and this error for sodium glutamate is large enough to explain what we have discovered about glutamate-glycine betaine interactions.

All of these reasons, as well as the observations and explanations given in the main article, show that solubility measurements for high solubility compounds are a poor choice for building a quantitative model of protein-osmolyte interactions. The data also show that multiple experimenters and labs can reproduce solubility measurements with high precision (e.g. leucine), so solubility measurements may be useful, with high precision instrumentation, for interactions of low solubility compounds with osmolytes (Guinn, E. J. et al., *Proc Natl Acad Sci U S A* **2011**, *108* (41), 16932–16937. <https://doi.org/10.1073/pnas.1109372108>). Carefully selected and reproduced solubility data, especially with low solubility compounds, may be useful inputs to quantitative models.

# Calculating ΔASA for side chains, backbone, and atoms

Calculating changes in accessible surface area for two structure files is quite straightforward with access to appropriate software. Several programs have been published that vary mostly in the speed with which they do the calculations. Because it is readily available online by the University of Texas Medical Branch, I used GetArea, as noted in Methods and Table 2. This gives as output accessible surface areas split up by backbone and side chains as well as by atoms. ΔASA is calculated by subtracting ASA values for corresponding side chains and adding up side chains of the same type. Or analogously by subtracting values for corresponding atoms and adding up values for the same atom type. Recognizing that this is easily reproduced by a researcher wishing to do similar calculations, and that GetArea is not my original research contribution, I have omitted the full data set. However, I have included a spreadsheet with the calculations for Ser-13 and Ser-14 that were used to create Table 3 as an example of the process.
